# Physical Surface Modification of Carbon-Nanotube/Polydimethylsiloxane Composite Electrodes for High-Sensitivity DNA Detection

**DOI:** 10.3390/nano11102661

**Published:** 2021-10-10

**Authors:** Junga Moon, Huaide Jiang, Eun-Cheol Lee

**Affiliations:** 1 Department of Nano Science and Technology, Graduate School, Gachon University, Seongnam-si 13120, Gyeonggi-do, Korea; mka3202@naver.com (J.M.); huaide20@gmail.com (H.J.); 2 Department of Physics, Gachon University, Seongnam-si 13120, Gyeonggi-do, Korea

**Keywords:** biosensor, physical surface modification, dip coating, functionalized carbon nanotube, electrochemical impedance spectroscopy

## Abstract

The chemical modification of electrode surfaces has attracted significant attention for lowering the limit of detection or for improving the recognition of biomolecules; however, the chemical processes are complex, dangerous, and difficult to control. Therefore, instead of the chemical process, we physically modified the surface of carbon-nanotube/polydimethylsiloxane composite electrodes by dip coating them with functionalized multi-walled carbon nanotubes (F-MWCNTs). These electrodes are used as working electrodes in electrochemistry, where they act as a recognition layer for sequence-specific DNA sensing through π–π interactions. The F-MWCNT-modified electrodes showed a limit of detection of 19.9 fM, which was 1250 times lower than that of pristine carbon/polydimethylsiloxane electrodes in a previous study, with a broad linear range of 1–1000 pM. The physically modified electrode was very stable during the electrode regeneration process after DNA detection. Our method paves the way for utilizing physical modification to significantly lower the limit of detection of a biosensor system as an alternative to chemical processes.

## 1. Introduction

Highly sensitive and selective detection of specific DNA sequences is crucial for biotechnological applications such as clinical diagnosis [1,2] and environmental [3,4] and food monitoring [5]. Therefore, extensive efforts have been devoted to developing techniques for detecting highly sensitive sequence-specific DNA sensors based on optical [6,7], electrical [8,9] and electrochemical [1,3,10,11] methods. Among these techniques, electrochemical methods have received considerable attention because they can be used to fabricate fast, simple, highly sensitive, and miniature DNA sensors [12,13]. Electrochemical impedance spectroscopy (EIS) is a very accurate electrochemical method, which is sensitive to changes on the electrode surface and is suitable for label-free DNA detection [14,15]. In several studies, EIS-based sensors have been fabricated using a gold electrode as the working electrode [16,17,18]. However, gold is very expensive and a laborious and inefficient process for immobilizing the probe is usually required for gold electrodes, which is unfavorable for commercialization.

Additionally, as an alternative to glassy carbon electrodes (GCEs), Au electrodes are extensively used as working electrodes in electrochemical DNA detection. However, because GCEs themselves do not recognize DNA molecules, chemical processes for immobilizing probes, which are more complicated than those for Au electrodes, are required when GCEs are used for electrochemical DNA detection [19,20,21]. Moreover, to further reduce the limit of detection (LOD), chemical surface modification can involve other nanomaterials, such as carbon nanotubes [22] and graphene [23]. In these cases, the chemical surface modifications consist of multiple steps that link probe DNA and nanomaterials to the electrode surfaces [24,25,26] and require several reagents. To avoid the complexity of chemical processes, in previous studies, multi-walled carbon nanotube (MWCNT)/polydimethylsiloxane (PDMS) composite electrodes were fabricated for DNA detection, and MWCNTs were used as the main material for the electrode [27,28]. In contrast to GCE, the electrode can behave as a recognition layer for DNA sensors without chemical modification; in addition, it is flexible and easy to fabricate. However, the LOD for DNA is 25 pM, which is higher (32–350 fM) than those of some chemically treated GCE-based sensors [29,30] and lower (38–275 pM) than those of other chemically treated GCE-based sensors [31,32].

In this study, to further decrease the LOD of a MWCNT/PDMS electrode, we modified the physical surface of MWCNT/PDMS composite electrodes using functionalized MWCNTs (henceforth called F-MWCNTs) using a dip-coating process. This electrode was used as the working electrode for the electrochemical DNA sensor. Compared to previous sensors based on pristine MWCNT/PDMS electrodes [27,28], the LOD decreased by approximately 1250 times, from 25 pM to 20 fM. The electrode was stably regenerated by ethanol and water cleaning, which indicated that the physical modification method was highly stable. Our results suggest that physical modification using F-MWCNT and dip-coating process could be a good alternative to chemical surface modifications for attaching nanomaterials to the electrode surfaces and that the F-MWCNT-modified electrode is a good alternative to expensive Au electrodes.

## 2. Materials and Methods

### 2.1. Materials and Reagents

The MWCNTs (diameter: 10–15 nm; length: 30–40 μm) were obtained from Hanwha Chemical (Daejeon, Korea) and the DNA samples, including probe DNA (P), complementary target DNA (T1), one base non-complementary target DNA (T2), and non-complementary target DNA (T3), were obtained from Bionics (Seoul, Korea) (Table 1). Nitric acid (HNO_3_, purity 69%) was obtained from Avantor (Radnor, PA, USA) and sulfuric acid (H_2_SO_4_, purity 98%) was obtained from Daejung (Siheung, Korea). The polydimethylsiloxane (PDMS) and potassium ferrocyanide (K_4_Fe(CN)_6_) were obtained from Sigma-Aldrich (St. Louis, MO, USA). The potassium ferricyanide (K_3_Fe(CN)_6_) was obtained from Junsei Chemical Co., Ltd. (Tokyo, Japan), membrane filters were obtained from Sigma-Aldrich (St. Louis, MO, USA) and 1 × phosphate-buffered saline (1 × PBS) solution (pH = 7.4) was obtained from Tech and Innovation Corporation (Chuncheon, Korea). Absolute ethanol was obtained from Fisher Scientific Inc. (Hampton, NH, USA) and isopropyl alcohol (IPA) solution was obtained from Sigma-Aldrich (St. Louis, MO, USA). The resistivity of deionized water (DI water) used throughout this study was 18.2 MΩ·cm. The Ag/AgCl reference electrode, with a potential of 0.197 V vs. SHE, and platinum wire, which was used as a counter electrode, was obtained from Princeton Applied Research (Oak Ridge, TN, USA). All other chemicals were of analytical reagent grade. To prepare a sample solution for the DNA detection experiments, target DNA with a specific concentration was added to the PBS solution containing 1000 nM probe DNA and 4 mM potassium ferrocyanide/potassium ferricyanide.

### 2.2. Instruments

All electrochemical methods, including EIS, cyclic voltammetry (CV), and differential pulse voltammetry (DPV) were measured using a CHI622D (CH Instruments, Inc., Austin, TX, USA). A tip sonicator (HD 2070, Bandelin sonopuls, Berlin, Germany) was utilized to disperse the MWCNT solvent. A dip coater (PTL-UMB, MTI Co., Richmond, CA, USA) was used to modify the MWCNT/PDMS electrode surface with the F-MWCNT solution and a UV-visible spectrophotometer (Cary 50, Varian, Mulgrave, Australia) was used to measure ultraviolet (UV) absorption spectroscopy. A scanning electron microscope (SEM; S4800, Hitachi, Tokyo, Japan) was used to obtain cross-sectional images and surface morphology of the F-MWCNT/MWCNT/PDMS electrodes and Fourier-transform infrared spectroscopy (FTIR) was used to obtain an infrared spectrum of transmittance of the F-MWCNT solution (L160000A, PerkinElmer, Waltham, MI, USA).

### 2.3. Procedures

#### 2.3.1. Preparation of Functionalized CNTs

**Figure 1:**
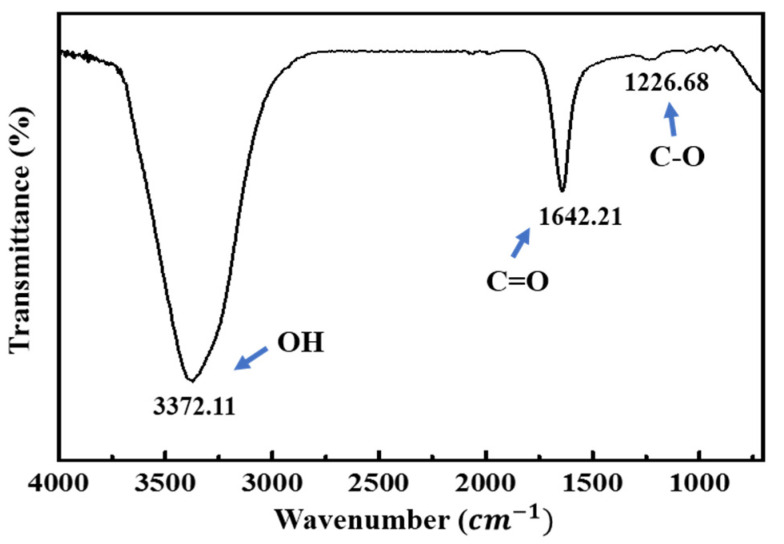
FTIR spectra of MWCNT modified with COOH.

The preparation of the functionalized CNTs is summarized in Figure S1a. First, the MWCNTs (0.2 g) were sonicated in a 3:1 (*v/v*) mixture of nitric acid and sulfuric acid solution [33]. Subsequently, the MWCNTs were filtered and rinsed with DI water using vacuum filtration until the pH of the filtrate was neutral. Finally, the filtrate was dried in an oven (Figure S1b) and sonicated for 5 min with 60 mL of DI water to make the F-MWCNT solution. As shown in Figure S1c, our F-MWCNTs were uniformly dispersed in DI water because they have better dispersion than MWCNTs in water [34]. The FTIR spectra (Figure 1), showed that there were O-H, C=O, and C-O vibrations at 3372.11, 1642.21, and 1226.28 cm^−1^, respectively, which clearly indicated that carboxylic and hydroxyl groups had been successfully attached to the surface of the MWCNTs.

#### 2.3.2. Process Method of the F-MWCNT/MWCNT/PDMS Electrode

**Figure 2:**
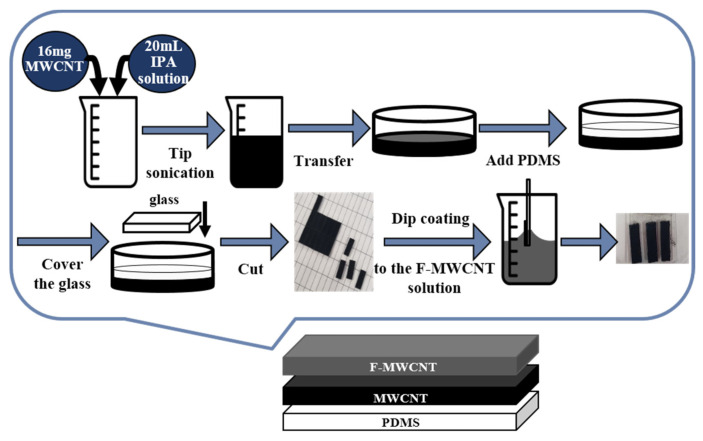
Fabrication process for the F-MWCNT/MWCNT/PDMS composite electrode.

A schematic representation of the technique used to fabricate the F-MWCNT/MWCNT/PDMS composite electrode is shown in Figure 2. First, the MWCNT composite electrode layer was fabricated according to a previous study’s methodology [28], which is explained in detail in Section 1 of the Supplementary Materials. Next, we coated the MWCNT/PDMS film with F-MWCNT solution according to the preparation method described above. Finally, the film was dip-coated at a dipping speed of 500 μm/s. In our experiments, the optimum repetition number for the same dip coating was found to be 60.

#### 2.3.3. Measurement Using EIS

Before the DNA detection experiment using EIS measurement, a sample solution was prepared by including one of the DNA targets (T1, T2, and T3) with a specific concentration of 1 nM DNA probe (P) and 4 mM [Fe(CN)_6_]^3−/4−^ in 1mL of PBS solution. The sample solvent was maintained at 25 °C for 7 min because the UV absorption of the sample solution was saturated after 7 min (Figure S2), which indicated that the DNA reaction in the solution had stabilized. After starting the EIS measurements, 9 min and 0.26 V were required to obtain the stabilized value of the charge transfer resistance (R_ct_) (Figure S3). Thus, a preparation time of 16 min was needed for EIS-based DNA detection in our experiments. Our three-electrode system, which consisted of an Ag/AgCl reference electrode, platinum counter electrode, and 5 mm × 5 mm F-MWCNT/MWCNT/PDMS working electrode, was placed in sample solutions, as shown in Figure S4. The EIS measurements were performed in the frequency range of 0.1 Hz to 10000 Hz, with an AC (alternating current) amplitude of 5mV and a DC (direct current) bias of 0.26 V for DNA detection.

## 3. Results and Discussion

### 3.1. SEM Measurments of the Composite Electrode

**Figure 3:**
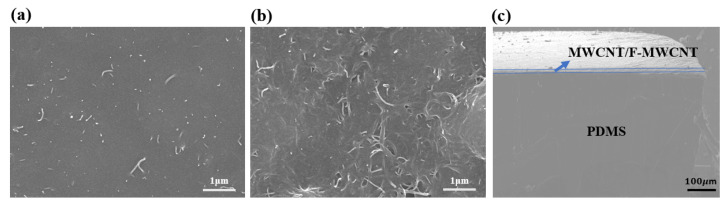
SEM image of the surface of MWCNT before (**a**) and after (**b**) the dip coating process with F-MWCNT solution. (**c**) Cross section of the F-MWCNT/MWCNT/PDMS layer.

The cross-section and surface morphology of the F-MWCNT-modified and pristine MWCNT/PDMS electrodes were characterized using SEM. For the pristine MWCNT/PDMS electrode, most MWCNTs were buried in PDMS and only a few tubular structures of MWCNTs were visible near the electrode surface, as shown in Figure 3a. After 60 dip-coating cycles with the F-MWCNT solution, many F-MWCNTs were observed on the surface of the F-MWCNT-modified MWCNT/PDMS electrode (see Figure 3b). These may act as active centers for DNA interactions. Figure 3c shows the cross-section of the F-MWCNT/MWCNT composite electrode. The results indicate that the mean thickness of the PDMS layers was approximately 430 μm. In addition, the mean thickness of the recognition layer was 15 μm, which increased by approximately 5 nm after the deposition of F-MWCNTs on the surface.

### 3.2. Characterization of the F-MWCNT-Modified Electrodes

**Figure 4:**
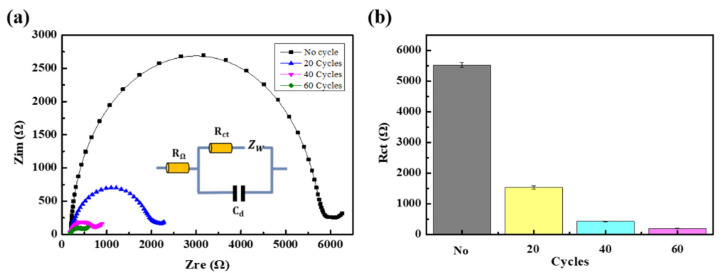
(**a**) Nyquist plots. (**b**) R_ct_ values before using dip coating and after dip coating forv20, 40, and 60 cycles.

Figure 4a showed how the EIS characteristic of the electrode was changed for different numbers of dip coating. R_ct_ for 0, 20, 40, and 60 dip coating cycles (Figure 4b) were obtained by fitting the Nyquist plots using F-MWCNT-modified electrodes in PBS solvent containing 4 mM [Fe(CN)_6_]^3−/4−^, with the equivalent circuit shown in Figure 4a. The circuit model is widely used for describing processes at the electrochemical interfaces [35,36], consisting of the active electrolyte resistance (R_Ω_), double-layer capacitance (C_d_), charge transfer resistance (R_ct_), and Warburg impedance (Z_w_). R_ct_ and Z_w_ describe the electron transfer and the mass transport of the electroactive species near the solution–electrode interface, respectively [37]. The R_ct_ of the film decreased when increasing the number of dip coating cycles, as shown in Figure 4b; as compared to R_ct_ for no dip coating (5500 Ω), that for 60 dip coatings (200 Ω) was about 27.5 times smaller. When the number of dip coatings was over 60, we found that the F-MWCNT/MWCNT layers were easily peeled off from the electrode during the electrochemical analysis. Thus, the number of dip coating cycles in the standard fabrication process was set to 60.

We think these results were obtained for two reasons. First, the charge transfer resistance might be associated with the number of MWCNTs on the electrode surface. In the MWCNT electrode, only a small number of MWCNTs and active area were exposed because some of the MWCNTs were buried in the PDMS. However, after depositing F-MWCNTs, the number of MWCNTs and the active area of the electrode were increased. Second, we think the energy barrier of the redox species reaching the electrode was lowered due to Coulomb or steric interactions [38]. We speculate that the energy barrier of [Fe(CN)_6_]^3−/4−^ approaching the electrode surface is lowered by hydrogen bonding affinity to carboxylic and hydroxyl groups in F-MWCNTs. 

**Figure 5:**
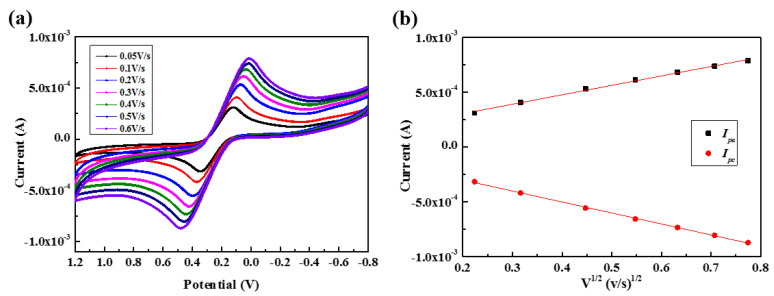
(**a**) Cyclic voltammograms of 4mM [Fe(CN)_6_]^3−/4−^ for a F-MWCNT/MWCNT/PDMS electrode with scan rates from 0.05 to 0.6 V/s and (**b**) anodic and cathodic peak currents (*I_pa_* and *I_pc_*, respectively) as a function of square root of the scan rates (ν).

Cyclic voltammetry (CV) is one voltammetry technique for measuring the current response of a redox active solution to a linearly cycled potential sweep. To investigate whether the reactions near the electrodes are diffusion-controlled, we performed CV measurements with F-MWCNT/MWCNT/PDMS electrodes for 4mM [Fe(CN)_6_]^3−/4−^ in the PBS solution, varying the scan rate from 0.05 to 0.6 V/s (see Figure 5a). Figure 5b indicates that the anodic and cathodic peak currents (*I_pa_* and *I_pc_*, respectively) increased linearly with the square root of the scan rate (ν). The linear equations can be described as; $I_{pa}\left(A\right)=\left(8.598\times 10^{-4}\right)\times \nu^{\frac{1}{2}}+1.311\times 10^{-4}$ with an $R^{2}=0.9953$ and $I_{pc}\left(A\right)=-0.001\times \nu^{\frac{1}{2}}-1.006\times 10^{-4}$ with an $R^{2}=0.9989$. These results indicate that the oxidation–reduction reactions were diffusion controlled, indicating that the composite electrode is appropriate for quantitative electrochemical analysis [39,40]. 

### 3.3. DNA Detection Using F-MWCNT-Modified Electrodes

**Figure 6:**
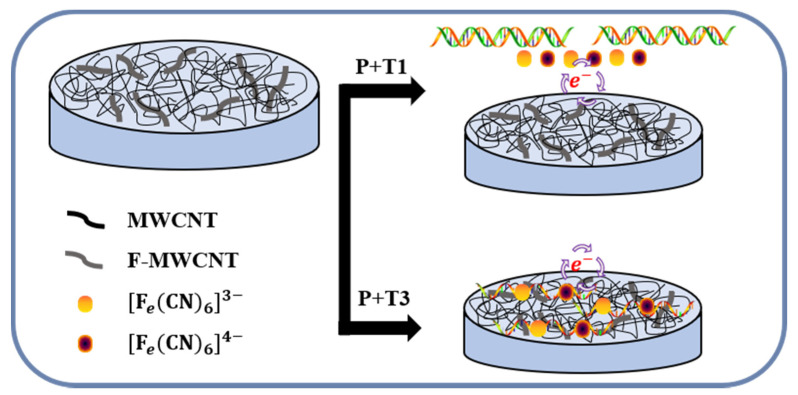
Schematic diagram of DNA detection mechanism.

As discussed above, a F-MWCNT/MWCNT/PDMS electrode could be used as a DNA sensor. The schematic of the DNA detection mechanism is described in Figure 6. It is well known that single-stranded DNA (s-DNA) is adsorbed on the surfaces of F-MWCNT or MWCNT through π–π interactions, whereas double-stranded DNA (d-DNA) is not [4,41,42,43,44,45]. For target T3 and probe DNA, the sequences of the probe and target are all mismatched, so that the single-stranded target and probe DNA might be adsorbed onto the recognition layer (F-MWCNT/MWCNT) of the electrode through π–π interactions [4,43,44]. The adsorbed s-DNA can act as the barrier of charge transfer between the oxidation–reduction couple ([Fe(CN)_6_]^3−/4−^) and the MWCNTs, increasing R_ct_, whereas for target T1 and probe DNA, d-DNA can form through hybridization between T1 and probe DNA because their sequences match completely. The vast majority of d-DNA remains far from the electrode rather than being adsorbed onto the recognition layer [45]. Thus, because d-DNA does not behave as a charge transfer barrier, R_ct_ is expected to be lower than that for the former case with s-DNA adsorbed on the electrode. In the case of T2, the probe and target DNA have a one-base mismatch. Because the level of sequence mismatch is between those for T1 and T3, resistance might also be between those of T1 and T3.

**Figure 7:**
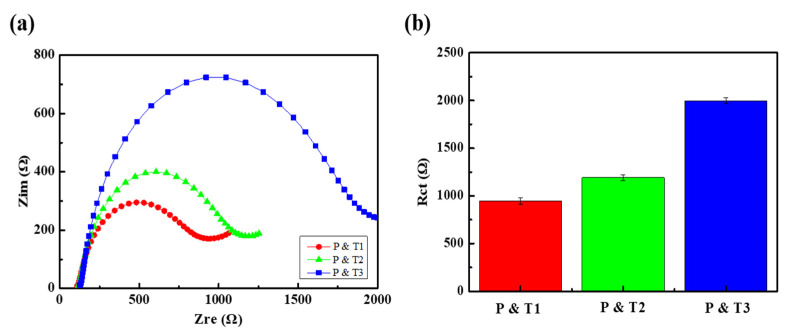
(**a**) Nyquist plots and (**b**) R_ct_ values for 1 nM probe DNA and 1 nM target DNA (T1, T2 and T3) in 4 mM [Fe(CN)_6_]^3−/4−^ solution with 1× PBS buffer (pH = 7.4).

We performed EIS measurements for the sample solutions described in the experimental section, which include probe DNA (P) and one target DNA (T1, T2, or T3), with the target as shown in Figure 7a. R_ct_ was calculated from the fitting of the Nyquist plots in Figure 7a, the values of which were about 940, 1190, and 2000 Ω for the T1, T2, and T3 targets, respectively, as shown in Figure 7b. The highest R_ct_ values were obtained for probe and T3, whereas the lowest were for probe and T1, as expected due to the mechanism discussed above. We defined the DNA detection sensitivity, г, by the equation г = ∆R/R°, where R° is the R_ct_ when hybridized with T3 and ∆R is the difference between the R_ct_ for the present target and R°. According to this equation, the sensitivities for perfectly matched and one-base mismatched targets (T1 and T2) were 52.5% and 40.4%, respectively; the sensitivity differs by 12.1% even for a one-base mismatch, indicating the good sequence selectivity of our system.

### 3.4. Detection Limit of the DNA Sensor

**Figure 8:**
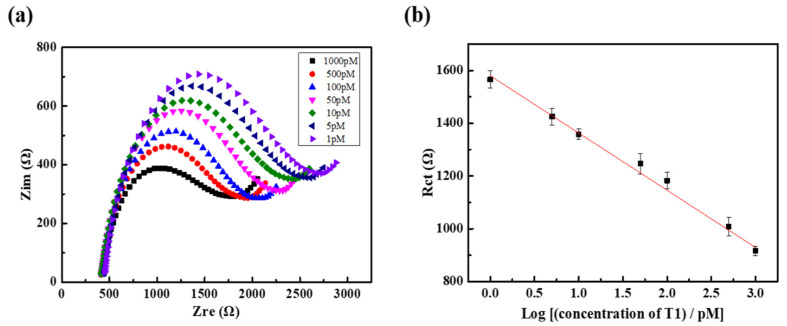
(**a**) Nyquist plots for the 1 nM probe DNA and various target (T1) concentrations and (**b**) plot of R_ct_ values against the logarithm of the various target (T1) concentrations.

As shown in Figure 8b, R_ct_ in the EIS analysis with target T1 was linearly dependent on the logarithm of the T1 concentration in the range of 1–1000 pM in the sample solutions. In Figure 8a, a decrease in the R_ct_ is clearly observed alongside an increase in the concentration of T1. The reason might be that as the concentration of T1 increases, the amount of DNA attached to the active site increases, which increases the current and reduces R_ct_. The linear regression equation was determined to be $R_{\mathrm{ct}}=-2.17\times 10^{2}\cdot logC_{1}+1.58\times 10^{3}\;$with an R^2^ = 0.992, where *C*_1_ is the concentration of T1 ranging from 1 to 1000 pM, as shown in Figure 8b. The LOD was extrapolated to be 19.9 fM using a signal-to-noise ratio of 3:1. Our LOD decreased by 1250 times compared to that obtained in a previous study, wherein a MWCNT electrode was used as a working electrode for DNA detection in EIS [27]. The 27.5 times reduction in R_ct_ by F-MWCNT modification to the MWCNT/PDMS electrode surface might be an important reason for the drastic decrease in LOD. The change in the adsorption/desorption characteristics of the s-DNA and d-DNA on the electrode surface by F-MWCNT modification may also have contributed to lowering the LOD. Furthermore, F-MWCNTs can interact with DNA through hydrogen bonding and electrostatic interactions, as well as π–π stacking interactions, and these interactions are more complicated than the interactions in MWCNTs. Although a comprehensive study of DNA adsorption characteristics on F-MWCNTs was beyond the scope of this study, it is known that graphene oxide, which has a chemical structure similar to that of F-MWCNT, absorbs s-DNA well, but does not efficiently absorb d-DNA [46]. Graphene-oxide-based DNA sensing can be performed in less than 30 min, whereas it takes several hours to achieve a similar detection rate with carbon nanotubes, which do not have functional groups [47]. Based on these results, we speculate that F-MWCNTs are more efficient for discriminating s-DNA and d-DNA than MWCNTs owing to their additional interactions, such as hydrogen bonding.

### 3.5. Stability of the Modified Electrode

**Figure 9:**
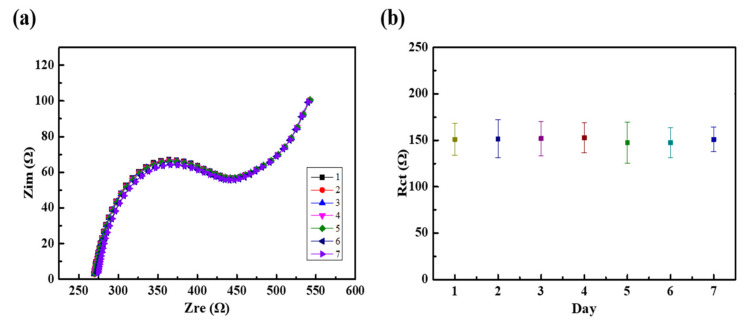
(**a**) Nyquist plots and (**b**) R_ct_ values for 1 nM probe DNA and target 1 DNA measured every 24 h for 7 days in 4 mM [Fe(CN)_6_]^3−/4−^ solution with 1× PBS buffer (pH = 7.4).

The stability of the fabricated DNA sensor over 7 days was investigated by measuring the average R_ct_ daily (Figure 9 and Figure S5). For the experiment, 2 mL of sample solvent containing 1 nM P and T1 DNA was poured into the modified electrode. It was first stored at room temperature for 1 week and then examined via its EIS response (Figure 9a) after hybridization in 4 mM [Fe(CN)_6_]^3−/4−^ solution with 1× PBS buffer. Between every experiment, the electrode was cleaned by ultrasonication in absolute ethanol for 3 min and DI water for 3 min. Figure 9b shows the charge transfer resistance (R_ct_) and the relative standard deviation of R_ct_ is 2.31%, indicating the good stability of the fabricated electrode. The CV curves of the fabricated electrode are shown in Figure S5 (one cycle and 100 cycles). Even after 100 cycles, the results show that there was no significant change. This indicates that our physical surface modification using F-MWCNTs is very stable during the regeneration process. It is important to note that our electrode method is not a chemical but a physical process; therefore, it is easy to control, thereby reducing LOD, and is applicable to other carbon sensors.

## 4. Summary

We investigated the physical surface modification of multi-walled carbon-nanotube/polymer electrodes treated using F-MWCNTs and a dip-coating process. The F-MWCNTs could be successfully deposited using solution-based physical processes, such as dip coating, because of their high dispersibility in water. The electrode surface can be used as a recognition layer, enabling the simple fabrication of DNA sensors. The vital properties of our structure are that by modifying it with F-MWCNTs, the active area of the electrode was increased, which resulted in a low charge transfer resistance. Compared to EIS-based DNA sensors that use electrodes chemically modified with nanomaterials, our electrode has a simpler and more controllable fabrication process with a very low LOD of 19.9 fM. The physically modified electrodes were stable during the regeneration process. Our results indicate that the physical surface modification of electrodes is a promising alternative approach to chemical surface. 

## Figures and Tables

**Table 1:** Probe and target nucleotide sequences. The bases leading to the mismatches are underlined.

Type	Name	Sequence
Probe DNA	P	5′-GTG TTG TCT CCT AGG TTG GCT CTG-3′
Complementary target DNA	T1	5′-CAG AGC CAA CCT AGG AGA CAA CAC-3′
One base-non-complementary target DNA	T2	5′-CAG AGC CAA CCT CGG AGA CAA CAC-3′
Non-complementary target DNA	T3	5′-ATA TCG ACC TTG GCC GAG ACG GTG-3′

## Data Availability

Data sharing is not applicable to this article.

## Supplementary Materials

The following are available online at https://www.mdpi.com/article/10.3390/nano11102661/s1, Figure S1: (a) Fabrication process for the functionalized MWCNT (MWCNT-COOH). (b) Membrane filter remaining after processing by vacuum filtration and (c) after sonicating with DI water.; Figure S2: Determination of hybridization time. (a) Sixteen UV absorption curves measured every 30 s for 480 s. (b) Absorbance at a wavelength of 260 nm as a function of time; Figure S3: (a) Nyquist plot for the different accumulation times at 0.26 V from 1 min to 15 min. (b) R_ct_ value as a function of time. The sample PBS solutions contained 1 nM probe DNA and 4 mM [Fe(CN)_6_]^3−/4−^.; Figure S4: (a) Schematic and (b) image showing the integration of the three electrodes platform.; Figure S5: Cyclic voltammetry responses of the F-MWCNT/MWCNT/PDMS electrode after 1 cycle and after 100 cycles. The electrode was immersed in PBS solution containing 4 mM [Fe(CN)_6_]^3−/4−^. Table S1: Fitted values of the equivalent circuit parameters from dark Nyquist plots of devices without and with 5% Cs.
