# Supplementary material for: Physical Surface Modification of Carbon-Nanotube/Polydimethylsiloxane Composite Electrodes for High-Sensitivity DNA Detection
Source: Nanomaterials (Basel). 2021 Oct 10;11(10):2661. doi: 10.3390/nano11102661 (PMC8541392; doi:10.3390/nano11102661)
Supplement: Supplementary file 1 [file nanomaterials-11-02661-s001.zip › nanomaterials-1394669 Supplementaryl.pdf]

nanomaterials

Supplementary Material

Physical Surface Modification of Carbon Nanotube/Polymer Electrodes for High-Sensitivity DNA Detection

Junga Moon [1,3], Huaide Jiang [1,3] and Eun-Cheol Lee [1,2,3,*]

1 Department of Nano Science and Technology, Graduate School. Gachon University, Gyeonggi 13120, Republic of Korea; mka3202@naver.com (J.M.); huaide20@gmail.com (H.J.)
2 Department of Physics, Gachon University, Gyeonggi 13120, Republic of Korea
3 Gachon Bio-Nano Research Institute, Gachon University, Gyeonggi 13120, Republic of Korea
* Correspondence: ecleel@gachon.ac.kr; Tel.: +82 31 750 8752

1. Preparation of the F-MWCNT/MWCNT/PDMS electrode

16 mg of MWCNT powder was dispersed in 20 mL anhydrous IPA through tip sonication for 1 h. The process for forming MWCNT layer on a petri dish with a diameter of 9 cm consisted of three cycles of casting and drying the solution. During the first cycle, 10 mL of the MWCNT solution was placed onto a petri dish and dried at 95 °C. During the second and the third cycles, 5 mL of the solution was casted and dried. 11 g of PDMS was prepared by mixing an elastomer and curing agent in a weight ratio of 10:1 and air bubbles in PDMS were removed using a vacuum pump. Then, PDMS was poured onto the dry MWCNT layer and was covered the glass substrate on MWCNT/PDMS layer at 95 °C for 10 h. After that, MWCNT/PDMS/glass was detached off from petri dish and then the MWCNT/PDMS layer was also peeled off from the glass. The MWCNT/PDMS film was cut to 15 mm × 5 mm dimension and cleaned by sonicating it in absolute ethanol and deionized water for 3 min and 2 min, respectively.

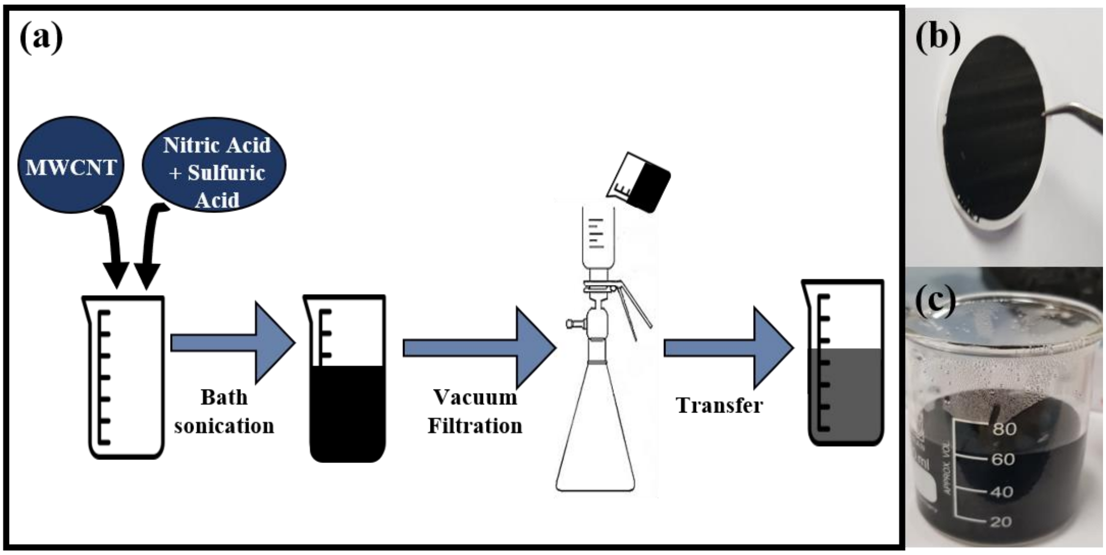

Figure S1. (**a**) Fabrication process for the functionalized MWCNT (MWCNT-COOH) (**b**) Membrane filter remaining after processing by vacuum filtration and (**c**) after sonicating with DI water.

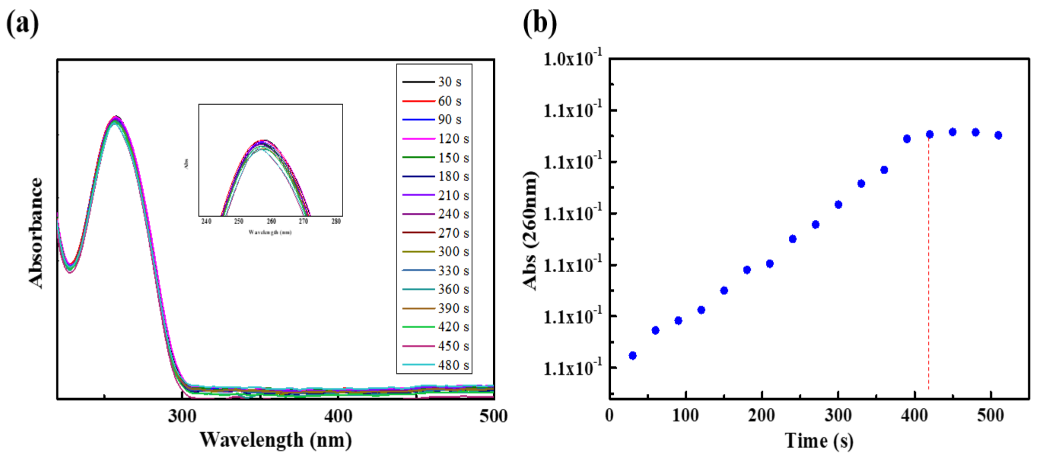

Figure S2. Determination of hybridization time. (**a**) Sixteen UV absorption curves measured every 30 s for 480 s. (**b**) Absorbance at a wavelength of 260 nm as a function of time.

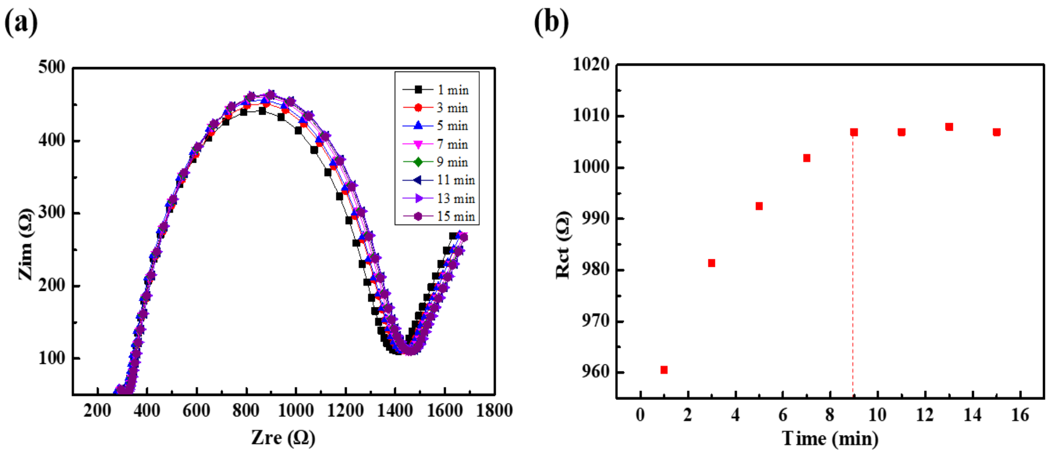

Figure S3. (**a**) Nyquist plot for the different accumulation times at 0.26 V from 1 min to 15 min. (**b**) R_{ct} value as a function of time. The sample PBS solutions contained 1 nM probe DNA and 4 mM $[Fe(CN)_6]^{3-/4-}$.

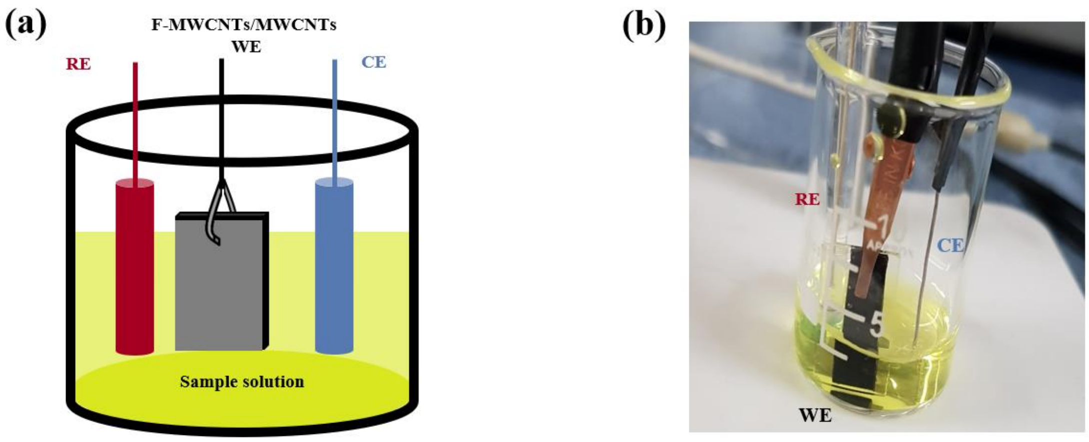

Figure S4. (**a**) Schematic and (**b**) image showing the integration of the three electrodes platform.

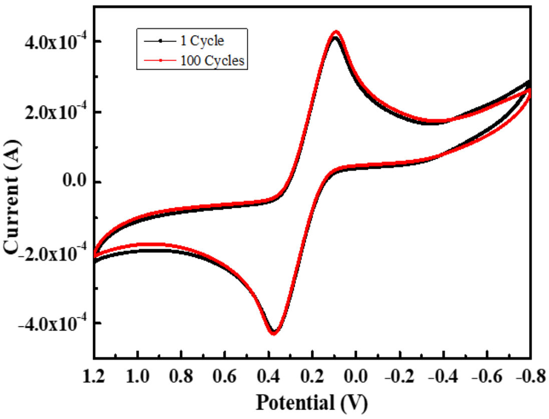

Figure S5. Cyclic voltammetry responses of the F-MWCNT/MWCNT/PDMS electrode after 1 cycle (black line) and after 100 cycles (red line). The electrode was immersed in PBS solution containing 4 mM $[Fe(CN)_6]^{3-/4-}$.

Table S1. Comparison of the electrode performance with other electrochemical DNA biosensors based on EIS.

Working electrode	LOD	Linear range (M)	References
GCE/AuNPs-ATP functionalized graphene oxide (ATP-GO)	11.3 fM	1.0×10^{-13} *to* 1.0×10^{-9}	[1]
GCE/electrochemically reduced graphene oxide (ERGO)	30 pM	1.0×10^{-12} *to* 1.0×10^{-9}	[2]
GCE/polyiline-mesoporous nanozirconia composite (PAN-nanoZrO_2)/ Poly L-tyrosine (PTyr)	26.8 fM	1.0×10^{-13} *to* 1.0×10^{-6}	[3]
Carbon paste electrode/nanogold–CNT/polyaniline nanofibers (PAN_{nano}) films	0.56 pM	1.0×10^{-12} *to* 1.0×10^{-6}	[4]
GCE/SWCNTs/ZrO_2/2,6-pyridinedicarboxylic acid (PDC)	1.38 pM	1.0×10^{-11} *to* 1.0×10^{-6}	[5]
Au electrode/AuNPs	0.67 pM	2.0×10^{-12} *to* 9.0×10^{-8}	[6]
F-MWCNT/MWCNT/PDMS composite electrode	19.9 fM	1.0×10^{-12} *to* 1.0×10^{-9}	This work

References

1. Gupta, V.K.; Yola, M.L.; Qureshi, M.S.; Solak, A.O.; Atar, N.; Üstündağ, Z. A novel impedimetric biosensor based on graphene oxide/gold nanoplatform for detection of DNA arrays. *Sensors Actuators, B Chem.* **2013**, *188*, 1201–1211, doi:10.1016/j.snb.2013.08.034.
2. Gong, Q.; Yang, H.; Dong, Y.; Zhang, W. A sensitive impedimetric DNA biosensor for the determination of the HIV gene based on electrochemically reduced graphene oxide. *Anal. Methods* **2015**, *7*, 2554–2562, doi:10.1039/c5ay00111k.
3. Yang, J.; Wang, X.; Shi, H. An electrochemical DNA biosensor for highly sensitive detection of phosphinothricin acetyltransferase gene sequence based on polyaniline- (mesoporous nanozirconia)/poly-tyrosine film. *Sensors Actuators, B Chem.* **2012**, *162*, 178–183, doi:10.1016/j.snb.2011.12.064.
4. Zhou, N.; Yang, T.; Jiang, C.; Du, M.; Jiao, K. Highly sensitive electrochemical impedance spectroscopic detection of DNA hybridization based on Aunano-CNT/PANnano films. *Talanta* **2009**, *77*, 1021–1026, doi:10.1016/j.talanta.2008.07.058.
5. Yang, J.; Jiao, K.; Yang, T. A DNA electrochemical sensor prepared by electrodepositing zirconia on composite films of single-walled carbon nanotubes and poly(2,6- pyridinedicarboxylic acid), and its application to detection of the PAT gene fragment. *Anal. Bioanal. Chem.* **2007**, doi:10.1007/s00216-007-1450-5.
6. Zhang, K.; Ma, H.; Zhang, L.; Zhang, Y. Fabrication of a sensitive impedance biosensor of DNA hybridization based on gold nanoparticles modified gold electrode. *Electroanalysis* **2008**, *20*, 2127–2133, doi:10.1002/elan.200804290.
